# Post-EVAR Endoleaks: A Morphovolumetric Approach to Prediction, Surveillance, and Management

**DOI:** 10.3390/jcm15114300

**Published:** 2026-06-02

**Authors:** Emre Külahcıoğlu, Sinan Özçelik, Nuh Can Koçak, Emre Çiçekyurt, Bekir Boğaçhan Akkaya, Bahadır Aytekin, Hakkı Zafer İşcan

**Affiliations:** 1 Department of Cardiovascular Surgery, Kilis Prof. Dr. Alaeddin Yavaşca Devlet Hastanesi, Kilis 79000, Turkey; 2 Department of Cardiovascular Surgery, Sincan Eğitim ve Araştırma Hastanesi, Ankara 06949, Turkey; drozceliksinan@gmail.com (S.Ö.); nuhcankocak@gmail.com (N.C.K.); 3 Department of Cardiovascular Surgery, Kulu Bölge Devlet Hastanesi, Konya 42780, Turkey; dremrecicekyurt@gmail.com; 4 Department of Cardiovascular Surgery, Ankara Şehir Hastanesi, Ankara 06800, Turkey; bullchan@gmail.cim (B.B.A.); bahadiraytekin@hotmail.com (B.A.); zafirustr@yahoo.com (H.Z.İ.)

**Keywords:** endoleak, EVAR, aortic aneurysm, endovascular, volumetric

## Abstract

**Background/Objectives**: To evaluate the association of preoperative morphometric and morphovolumetric parameters with post-endovascular aneurysm repair (EVAR) sac remodeling, endoleak development, and secondary interventions, and to assess the role of volumetric analysis in post-EVAR surveillance. **Methods**: This retrospective single-center study included 383 patients who underwent elective EVAR for infrarenal abdominal aortic aneurysm between 2016 and 2024, with available pre- and postoperative computed tomography angiography and at least 1 year of follow-up. Diameter- and volume-based sac dynamics were analyzed using standardized morphometric and 3-dimensional morphovolumetric measurements. Endoleak subtype distribution, risk factors, secondary interventions, and survival were assessed using regression and survival analyses. **Results**: Endoleaks were detected in 26.1% of patients (*n* = 100), with type II endoleak being the most frequent subtype (12.3%, *n* = 47), followed by type Ib (6.8%, *n* = 26), type III (5.5%, *n* = 21), type Ia (4.2%, *n* = 16), and 1 patient with type V endoleak in the revised manuscript framework. Secondary interventions were required in 14.1% of patients (*n* = 54), mainly for type I and III endoleaks, with a mean time to reintervention of 21.7 ± 10 months. Diameter and volume changes were strongly correlated; a 10% increase in aneurysm volume corresponded to an average 4 mm increase in diameter (R^2^ = 0.72, *p* < 0.001). Significant predictors of overall endoleak included dual antiplatelet therapy, aneurysm length > 133 mm, elevated pre- and postoperative D-dimer levels, aneurysm diameter > 59 mm, aneurysm volume > 164 cm^3^, and thrombus volume > 89 cm^3^. Subtype-specific analyses identified distinct risk profiles for type Ia, Ib, II, and III endoleaks. Overall survival did not differ significantly between patients with and without endoleaks (*p* = 0.227), although worse survival was observed in type Ia and III endoleaks than in type II and Ib endoleaks. **Conclusions**: Preoperative morphovolumetric parameters are significant predictors of post-EVAR endoleaks and secondary interventions. Volumetric analysis may provide a complementary early signal of aneurysm sac remodeling beyond conventional diameter-based assessment, particularly in patients with type II endoleaks. However, the proposed volumetric thresholds remain exploratory and require prospective external validation before routine clinical adoption. Post-EVAR management should integrate endoleak subtype, sac behavior, and patient-specific morphovolumetric risk factors to improve surveillance and treatment selection.

## 1. Introduction

Endovascular aneurysm repair (EVAR) has revolutionized the management of infrarenal abdominal aortic aneurysms (AAA), providing a less invasive alternative to open surgical repair (OSR), with reduced perioperative morbidity and mortality. Randomized controlled trials (RCTs), including EVAR-1, DREAM, OVER, and ACE, have demonstrated favorable early outcomes with EVAR, such as lower 30-day mortality and faster recovery, leading to its widespread adoption for anatomically suitable patients [1,2,3]. Advances in graft technology, including new-generation materials and active fixation systems, have expanded anatomical applicability, improved sealing, and reduced complications [1,2,3,4,5].

EVAR offers several advantages over OSR, including shorter hospital stays, decreased blood loss, faster recovery, and lower early mortality [3]; however, long-term survival rates tend to converge [2,6]. EVAR is particularly advantageous in elderly or comorbid patients at high risk for open surgery [7,8]. Its minimally invasive nature allows treatment of a broader patient population, including those with significant comorbidities [9,10].

New generation stent-grafts improved conformability, sealing and fixation therefore reduced complications such as endoleaks and graft migration. These facts allowed tailored approaches to complex anatomies [4,5]. Active fixation systems, including EndoAnchors and EndoStaples, mechanically attach the graft to the aortic wall, enhancing the proximal seal and minimizing the risk of migration, especially in hostile neck anatomy (short, angulated, conical necks) [2,4,11]. These devices demonstrate high technical success and durable outcomes in preventing type I endoleaks. Low-profile delivery systems and improved materials have expanded the applicability of EVAR to patients with challenging iliac anatomy and smaller access vessels [4,5].

Endoleaks are persistent blood flow outside the endograft but within the aneurysm sac, the most common complication after EVAR, with incidence rates reported between 15% and 30% [4,5,7,12,13]. They are classified into five types based on origin and pathophysiology:

Endoleaks remain the most frequent EVAR-specific complication and are classified according to the source of persistent aneurysm sac perfusion [5,7,8]. Type I and Type III endoleaks are generally considered high-pressure leaks requiring prompt treatment because of direct sac pressurization and rupture risk [5,7,8,12]. Type II endoleaks, usually caused by retrograde collateral flow from lumbar arteries or the inferior mesenteric artery, are the most common subtype and are often managed conservatively unless associated with sac enlargement or persistence [7,12,13]. Type IV and Type V endoleaks are less common in contemporary practice [7,12]. Although management is primarily guided by endoleak type and sac behavior, reliable prediction of post-EVAR sac remodeling and secondary intervention remains challenging [8,10,12].

Despite significant advances in endovascular technology and imaging modalities, the optimal management of endoleaks remains a subject of ongoing debate. In particular, the management of T2EL varies widely across institutions, reflecting uncertainty regarding treatment thresholds and optimal intervention strategies.

The primary tool for EVAR surveillance was CTA. CDUS was also a surveillance tool for our patient cohort; however, for this study, morphovolumetric analysis was performed, and volumetric or conventional diameter measurements were utilized for sac Dynamics [14,15]. Consequently, the 2-phase of (venous and arterial phase) CTA was considered the preferred approach to identify endoleaks for post-EVAR follow-up.

Sex-related differences after EVAR have received increasing attention in contemporary vascular surgery literature. Recent studies suggest that female patients may present with less favorable proximal neck anatomy, smaller access vessels, greater anatomical complexity, and potentially higher rates of adverse events, type Ia endoleaks, secondary procedures, reinterventions, or worse long-term outcomes after EVAR [16,17]. In the present cohort, however, female patients represented only 7.0% of the study population. Therefore, the study was underpowered to perform a reliable sex-stratified outcome analysis, and sex-specific conclusions could not be drawn. This issue should be addressed in larger multicenter cohorts with adequate female representation.

Currently, there is no universally accepted, clinically applicable framework that integrates anatomical features, hemodynamic behavior, and aneurysm sac dynamics into a structured decision-making process. Therefore, this study aims to provide a comprehensive overview of endoleak management and propose a practical algorithm to guide treatment selection in clinical practice, however our study does not go in depth to describe each treatment.

## 2. Materials and Methods

Patients who underwent elective endovascular aneurysm repair (EVAR) for infrarenal abdominal aortic aneurysms (AAA) between 2016 and 2024 at our single tertiary medical center were retrospectively evaluated. Inclusion criteria comprised availability of both preoperative and postoperative contrast-enhanced computed tomography angiography (CTA) and a minimum follow-up duration of one year. A total of 383 patients met these criteria. Ethical approval was obtained from the Health Sciences University Ankara Bilkent City Hospital Medical Research Scientific and Ethical Review Board (decision no.: TABED 1-25-1189, dated 9 April 2025).

During the study period, 383 patients who underwent elective EVAR for fusiform infrarenal abdominal aortic aneurysm and had available preoperative and postoperative contrast-enhanced CTA with at least 1 year of follow-up were included in the final analysis. Patients with ruptured AAA requiring emergency intervention, juxtarenal AAA, complex endovascular procedures, concomitant TEVAR, incomplete or unavailable preoperative/postoperative CTA data, and follow-up shorter than 1 year were excluded. Because the retrospective database was constructed from patients meeting the final imaging and follow-up eligibility criteria, the exact number of patients excluded for each individual criterion could not be reconstructed reliably. All procedures were performed by the same surgical team.

EVAR procedures were performed by three vascular surgeons, of whom one were senior vascular surgeon with extensive experience in aortic endovascular procedures.

The primary endpoints of the study were to assess the impact of morphological and morphovolumetric parameters on aneurysm sac diameter and volume changes following EVAR, as well as their association with endoleak development. Secondary endpoints included rates and causes of secondary interventions, mortality, and morbidity post-EVAR.

Data collection was categorized into preoperative, intraoperative, postoperative, and follow-up phases. Preoperative data encompassed patient demographics, ejection fraction, history of prior abdominal surgeries, CTA-based measurements including aneurysm sac diameter, presence and localization of thrombus within the sac and neck, neck calcification, iliac tortuosity, and presence of iliac aneurysms. After preoperative D-dimer values were obtained, postoperative D-dimer was defined as the first D-dimer value measured within 24 h after EVAR. Three-dimensional (3D) total aneurysm sac volume, thrombus volume, and thrombus density were quantified. Intraoperative data included fluoroscopy times, contrast volumes used, and the occurrence of Type 1 endoleaks during or immediately after the procedure. Postoperative data comprised intensive care and ward stay durations, complications, length of hospitalization, and early mortality. Follow-up data included serial CTA measurements of aneurysm diameter, 3D total volume, thrombus volume and density, presence of endoleaks, secondary interventions, and late mortality. All patients were enrolled in a standardized postoperative surveillance protocol following EVAR. Follow-up evaluations were systematically conducted at 1 month, 6 months, and 12 months after the index procedure. At each time point, patients underwent clinical assessment and contrast-enhanced computed tomography angiography (CTA) using the same acquisition protocol as the preoperative imaging. Follow-up imaging focused on aneurysm sac diameter and volumetric changes, thrombus characteristics, graft integrity, and detection and classification of endoleaks. In addition, secondary interventions and late complications were recorded. In patients with contraindications to contrast agents, duplex ultrasonography was used as an alternative modality. This structured follow-up allowed consistent longitudinal evaluation of aneurysm sac remodeling and post-EVAR complications.

Postoperatively, all patients received β-blockers and Acetyl Salicylic Acid (ASA). In cases with external iliac artery extension, severe iliac tortuosity or significant atherosclerotic changes, dual antiplatelet therapy (ASA and clopidogrel) was initiated.

### 2.1. CTA Measurements

Preoperative and postoperative CTA-based diameter and angle measurements were performed using 3MENSIO Vascular software (Bilthoven, The Netherlands, version 10.6) and the Sarus Workstation connected to the Ankara City Hospital database. Coronal, sagittal, axial, and 3D reconstructions were analyzed, and data were exported to Excel. The anatomical and morphovolumetric variables analyzed in this study included maximum aneurysm diameter, aneurysm length, proximal neck diameter, proximal neck length, proximal neck morphology, neck thrombus, neck calcification, aortic neck angulation, iliac tortuosity, iliac artery aneurysm involvement, inferior mesenteric artery diameter, number of patent lumbar arteries, total aneurysm sac volume, patent lumen volume, sac thrombus volume, thrombus-to-aneurysm volume ratio, and thrombus localization. In this study, aortic angulation was defined according to the angle measured between the infrarenal proximal neck axis and the aneurysm sac axis on centerline-reconstructed CTA images. Although this measurement was referred to as the alpha angle in our institutional dataset, it corresponds to the neck–sac angulation used in EVAR planning and is conceptually similar to the beta angle definition used in some previous EVAR studies. To avoid ambiguity, the revised manuscript refers to this parameter as infrarenal neck–sac angulation.

3D morphovolumetric measurements were conducted by a cardiovascular surgeon and a radiologist with 10 years of experience using the Sarus Workstation. Volumes were measured in cubic centimeters (cm^3^), and densities in Hounsfield units (HU), from the proximal, mid, and distal aneurysm segments; the average of these three measurements was recorded. CTA scans were acquired with 128- and 512-detector CT scanners (GE Healthcare) following intravenous administration of 120 mL contrast at 4 mL/s during the arterial phase (20 s). Images were reviewed in axial, coronal, and sagittal planes, and semi-automated volumetric software (Advantage Workstation 4.2, GE Healthcare Technologies) was used to delineate the aneurysm sac outer wall circularly at the widest diameter to calculate diameter and volume. Thrombus volume refers to intraluminal thrombus volume within the aneurysm sac and was calculated by subtracting patent lumen volume from total aneurysm sac volume. This variable is distinct from proximal neck thrombus, which was analyzed separately as a sealing-zone characteristic.

Although measurements were performed using standardized semi-automated software by experienced readers, formal interobserver and intraobserver reproducibility testing was not performed. Therefore, the proposed volumetric thresholds, including the >164 cm^3^ cutoff, should be interpreted as exploratory. Segmentation variability may influence volumetric measurements, particularly in aneurysm sacs with extensive mural thrombus or circumferential calcification. These thresholds require prospective external validation before being used as definitive clinical decision cutoffs.

Preoperative and postoperative CTA measurements were performed with identical protocols.

Aneurysm sac dynamics post-EVAR were primarily monitored via changes in diameter and volume, which are the most widely accepted parameters. The Society for Vascular Surgery (SVS) defines significant sac growth as an increase in diameter of ≥5 mm or volume increase of ≥5%. Literature frequently uses a 5 mm threshold for diameter change and volume thresholds ranging from 5% to 10%. In this study, linear regression analysis demonstrated that a 10% increase in aneurysm volume corresponded to a 4 mm increase in diameter; thus, a 10% volume increase was adopted as the cutoff for positive remodeling.

**Figure 1:**
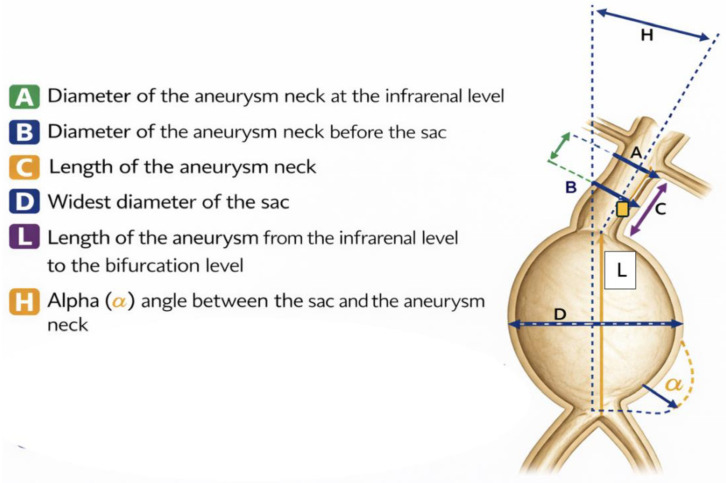
Morphometric parameters of Infrarenal Aortic Aneurysm.

Sac size changes were classified as follows: an increase in maximum diameter ≥ 5 mm was defined as “expanding sac,” a decrease ≥5 mm as “shrinking sac,” and changes within these limits as “stable sac.” Similarly, volumetric analysis categorized >10% decrease as “shrinking sac,” >10% increase as “expanding sac,” and changes between these values as “stable sac” (Figure 1).

### 2.2. Statistics

Data analysis was performed using IBM SPSS Statistics version 27.0 (IBM Corp., Armonk, NY, USA) and MedCalc version 15.8 (MedCalc Software, Ostend, Belgium). Continuous variables were summarized as mean ± standard deviation or median with range according to distribution, and categorical variables were summarized as frequency and percentage. Normality was assessed using the Kolmogorov–Smirnov test, skewness–kurtosis values, and graphical methods, including histograms and Q-Q plots.

Normally distributed continuous variables were compared using the independent-samples *t*-test, whereas non-normally distributed variables were compared using the Mann–Whitney U test. Categorical variables were compared using the chi-square test or Fisher’s exact test, when appropriate. Receiver operating characteristic (ROC) curve analysis was used to explore potential cutoff values for morphometric and morphovolumetric variables.

Logistic regression analyses were performed to evaluate factors associated with endoleak development. Variables were first assessed using univariate logistic regression. Variables with *p* < 0.10 in univariate analysis and variables considered clinically relevant based on previous literature and biological plausibility were considered for multivariable analysis. Backward stepwise selection was used in the subtype-specific multivariable models. To reduce the risk of overfitting, the number of variables entered into the models was restricted according to the number of outcome events, and subtype-specific analyses were interpreted as exploratory because of the limited number of events in individual endoleak categories.

Because aneurysm diameter, aneurysm volume, thrombus volume, and aneurysm length are anatomically related variables, potential collinearity was considered when constructing multivariable models. Highly correlated variables were not entered simultaneously into the same model when this could produce model instability. Optimal cutoff values in ROC analysis were selected using the Youden index.

Patients with incomplete preoperative or postoperative CTA data were excluded according to the predefined exclusion criteria. Missing data in the analytical dataset were handled by complete-case analysis, and no multiple imputation was performed. Survival was assessed using Kaplan–Meier analysis and compared using the log-rank test. Statistical significance was set at *p* < 0.05.

## 3. Results

A total of 383 patients undergoing elective EVAR for iAAA were retrospectively analyzed. The cohort was predominantly male (93%) with a mean age of 71.8 ± 7.8 years. Comorbidities were common, with hypertension (82%), smoking history (70%), and coronary artery disease (53%) being most prevalent. The mean proximal neck length was 25.7 ± 9.9 mm, and the mean proximal neck diameters were 23.0 ± 3.4 mm at level A and 23.7 ± 3.8 mm at level B. A conical neck configuration, defined as ΔA–B ≥ 3 mm, was present in 17.0% of patients. The mean infrarenal neck–sac angulation was 28.9 ± 23.4°. Neck thrombus and neck calcification were present in 30.8% and 18.3% of patients, respectively. Iliac tortuosity was present in 31.6% of patients. Endoleaks were detected in 26.1% (*n* = 100) of the patients during follow-up. All included patients had at least two postoperative CTA examinations. Duplex ultrasonography was used as an adjunctive follow-up modality but not as the sole imaging method for inclusion in the morphovolumetric analysis. Baseline and perioperative variables according to overall endoleak status is shown in Table 1.

Distrubition of the endoleak subtypes were;

- Type II: 12.3% (*n* = 47)—most frequent subtype, primarily arising from lumbar arteries and inferior mesenteric artery (IMA).
- Type Ib: 6.8% (*n* = 26)
- Type III: 5.5% (*n* = 21)
- Type Ia: 4.2% (*n* = 16)
- Type V: 0.3% (*n* = 1)

Because some patients developed more than one endoleak subtype during follow-up or had combined endoleak patterns requiring treatment in the same session, the sum of subtype counts exceeds the total number of patients with endoleak. Subtype percentages therefore represent the frequency of each endoleak subtype in the overall cohort, not mutually exclusive patient groups.

The mean radiological follow-up duration was 33.2 ± 21.2 months, with a median of 25 months and a range of 12–99 months.

Univariate logistic regression was performed to evaluate factors associated with overall endoleak development. The analysis included clinically relevant demographic, anatomical, morphovolumetric, laboratory, and treatment-related variables. Odds ratios, 95% confidence intervals, and exact *p*-values are presented in Table 2.

Multivariable logistic regression analyses were performed for endoleak subtypes with available model data. Because the number of events was limited for individual endoleak subtypes, these models were considered exploratory. The final multivariable models for Type Ia and Type II endoleaks are presented in Table 3. In the Type Ia model, hostile proximal neck features, including conical neck morphology, increased infrarenal neck–sac angulation, neck thrombus, and neck calcification, remained independently associated with Type Ia endoleak. In the Type II model, dual antiplatelet therapy, larger inferior mesenteric artery diameter, lower thrombus-to-aneurysm volume ratio, and anterior thrombus localization remained associated with Type II endoleak. These results should be interpreted cautiously because of the retrospective design and limited event numbers.

Multiple endoleak types were observed concurrently in a subset of patients during follow-up. In some cases, more than one endoleak subtype required simultaneous treatment within the same intervention session. Due to overlapping classifications and retrospective data structure, a precise numerical breakdown of combined endoleak patterns (e.g., dual or triple combinations) could not be consistently determined.

### Mean Time to Endoleak Diagnosis and Intervention by Endoleak Type

Secondary interventions were necessary in 14.1% (*n* = 54) of patients, mainly for Type I and III endoleaks, reflecting their higher rupture risk and clinical significance. The mean time to secondary intervention was 21.7 ± 10 months. Among these:

- Type Ia endoleaks (*n* = 16): Type Ia endoleaks (*n* = 16) were predominantly managed with endovascular techniques, in accordance with current EVAR guidelines recommending prompt treatment due to their direct systemic pressurization of the aneurysm sac [5,6].
- Type Ib endoleaks (*n* = 26): All treated with iliac extensions.
- Type III endoleaks (*n* = 21): 16 treated endovascularly, 1 with open surgery, 3 untreated due to refusal or clinical status.

**Figure 2:**
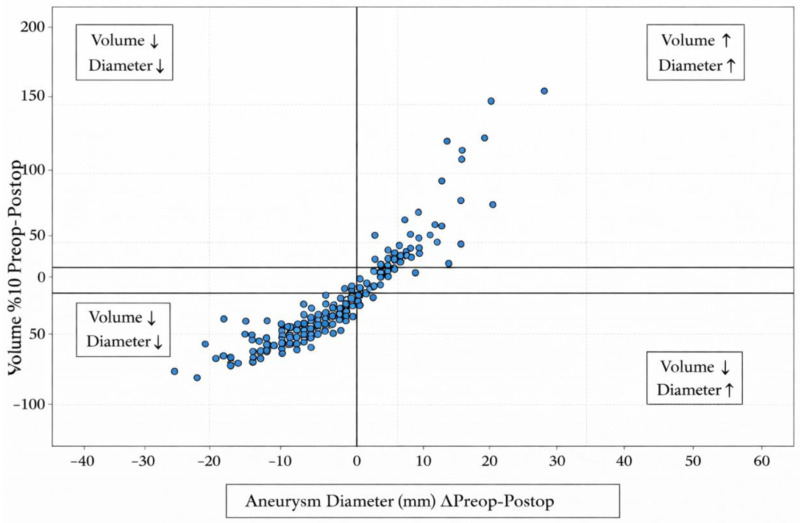
Scatter plot showing the relationship between aneurysm diameter and volume changes. Relationship between aneurysm sac volume change and maximum diameter change after EVAR. The fitted regression line with 95% confidence band is shown. The dashed vertical line indicates the 10% volumetric threshold, and the dashed horizontal line indicates the 5 mm diameter threshold.

**Figure 3:**
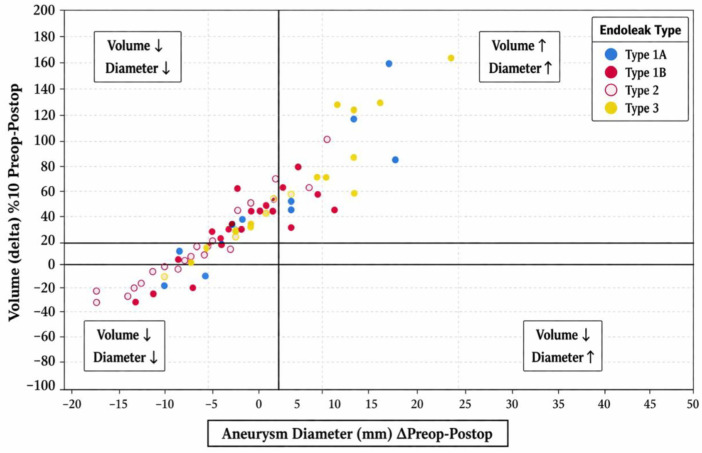
Distribution of sac diameter and volume changes by endoleak type. Sac remodeling according to endoleak subtype using diameter- and volume-based criteria. Threshold annotations indicate >=5 mm diameter enlargement and >10% volume increase. Type V endoleak is not used for comparative inference because only one patient was identified.

Volumetric and diameter analyses demonstrated a strong correlation (R^2^ = 0.72, *p* < 0.001) where a 10% change in aneurysm volume corresponded to an average 4 mm change in diameter. Volumetric measurements detected sac changes earlier than diameter alone, particularly in stable sacs and type II endoleaks, underscoring the sensitivity of volumetric assessment in endoleak monitoring (Figure 2 and Figure 3).

Regression analyses are summarized in Table 2 and Table 3. Overall endoleak development was associated with anatomical complexity, larger aneurysm dimensions, morphovolumetric parameters, elevated D-dimer levels, and dual antiplatelet therapy. However, because the study was retrospective and treatment allocation was not randomized, these findings should be interpreted as associative and exploratory.

**Endoleak Subtype-Specific Risk Profiles**

**Type Ia Endoleak**: Associated with advanced age, short neck length, conical neck morphology, infrarenal neck–sac angulation > 55°, neck calcification, and neck thrombus presence.

- **Type Ib Endoleak**: Correlated with peripheral arterial disease, aneurysm length > 133 mm, and presence of iliac artery aneurysms
- **Type II Endoleak**: Risk factors included dual antiplatelet therapy, IMA diameter > 3 mm, presence of ≥4 patent lumbar arteries, thrombus ratio to aortic lumen (TR/AO), and posterior thrombus localization. These parameters reflect the importance of collateral vessel anatomy and thrombus characteristics in type II endoleak pathophysiology.
- **Type III Endoleak**: Linked to larger aneurysm dimensions, thrombus volume, iliac tortuosity, and iliac aneurysm presence. These findings highlight mechanical stresses and graft component integrity as key factors in type III endoleak development.

At follow-up, 74.7% of patients exhibited aneurysm sac diameter reduction, 18.5% had stable sac size, and 6.8% experienced sac enlargement. Volumetric analysis revealed more sensitive detection of sac expansion, particularly in type II endoleaks.

**Figure 4:**
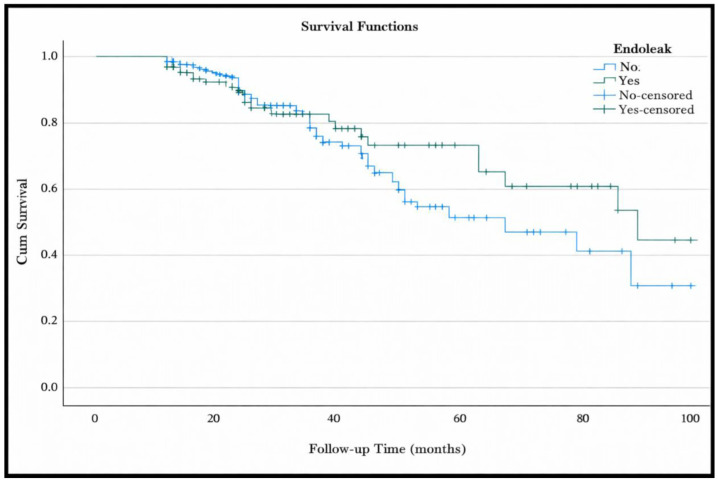
Kaplan–Meier survival curves by endoleak presence.

**Figure 5:**
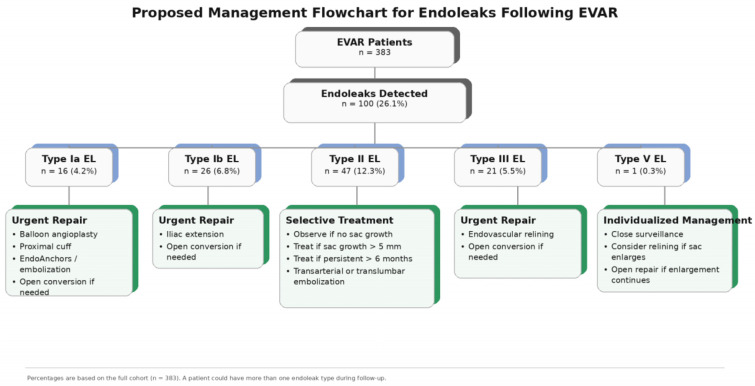
Proposed Management Flowchart for Endoleaks Following EVAR. (The Type V/endotension pathway is consensus-based and not cohort-derived).

Kaplan–Meier analysis showed no statistically significant difference in overall survival between patients with and without endoleaks (log-rank *p* = 0.227). Because subtype-specific survival curves were limited by small event numbers, particularly for Type Ia, Type III, and Type V endoleaks, subtype survival findings should be interpreted cautiously (Figure 4). Based on current evidence and clinical practice, we propose a structured decision-making framework for the management of endoleaks following EVAR (Figure 5).

Type I and Type III endoleaks require urgent intervention due to direct pressurization of the aneurysm sac. Initial management includes balloon angioplasty and extension cuff placement, with consideration of endoanchors or embolization in refractory cases.

T2ELs should be managed selectively. In cases without aneurysm sac expansion, conservative surveillance is appropriate. Intervention is recommended in patients with sac enlargement greater than 5 mm, persistent endoleaks beyond 6 months, or complex inflow-outflow vessel anatomy.

For treatment, transarterial embolization is preferred in patients with accessible feeding vessels, whereas translumbar embolization should be considered in cases of unfavorable vascular anatomy or prior failed interventions.

**Graph 1:**
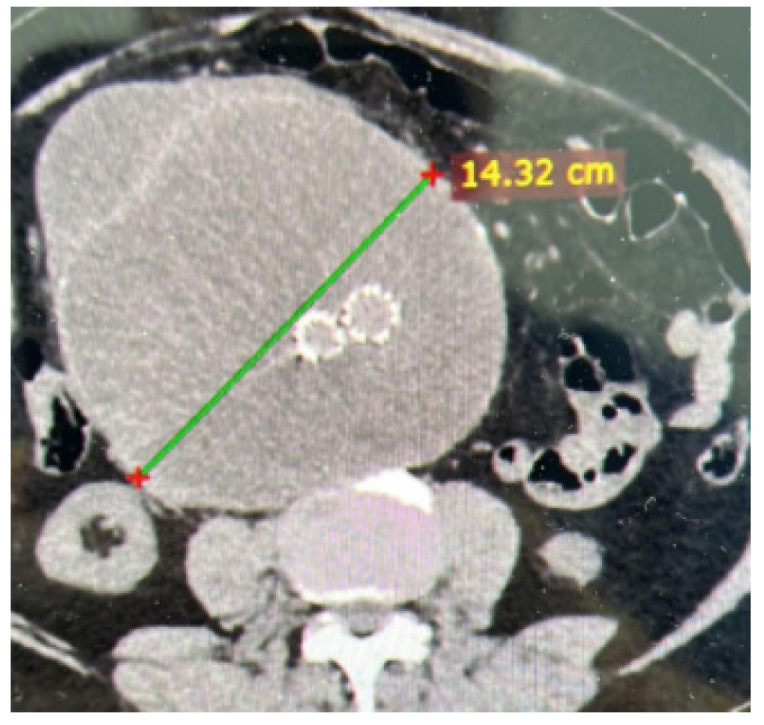
Type V endoleak.

Endotension (Type V) remains a diagnostic and therapeutic challenge, with management strategies ranging from observation to endograft relining or open surgical conversion depending on clinical progression. There was only one patient in our cohort with a documented type V endoleak who declined surgical intervention, likely due to frailty and advanced age. This patient presented with aneurysm rupture and died 11 months later. (Graph 1). Because only one Type V endoleak was observed in this cohort, no cohort-derived inference can be made for this subtype. Therefore, the Type V/endotension pathway in the proposed management algorithm is based on guideline-informed consensus and literature rather than on the present cohort data (Supplementary Materials).

## 4. Discussion

The management of endoleaks continues to represent one of the most complex and debated aspects of EVAR follow-up. While the need for immediate intervention in Type I and III endoleaks is well established due to direct aneurysm sac pressurization, significant controversy persists regarding the optimal management of T2ELs, particularly in asymptomatic patients without sac expansion. In our cohort, T2ELS were the predominant subtype encountered (12.3%), yet only a limited number of patients (*n* = 2) required secondary intervention, while a substantial proportion (53.2%) resolved spontaneously during follow-up. These findings suggest that most Type II endoleaks have a relatively benign natural course and may not necessitate immediate intervention. In contrast, Type I and Type III endoleaks were managed with early intervention due to their direct hemodynamic impact and associated risk of persistent aneurysm sac pressurization and rupture. Therefore, our results support a management strategy in which treatment decisions are guided primarily by aneurysm sac behavior and endoleak type, rather than the mere presence of an endoleak.

Sex-related differences after EVAR have received increasing attention in contemporary vascular surgery literature. Female patients may present with less favorable proximal neck anatomy, smaller access vessels, greater anatomical complexity, and potentially higher rates of adverse events, endoleaks, reinterventions, or worse long-term outcomes after EVAR [6,7,8,9,10]. In the present cohort, however, female patients represented only 7.0% of the study population. Therefore, the study was underpowered to perform a reliable sex-stratified outcome analysis, and sex-specific conclusions could not be drawn. This issue should be addressed in larger multicenter cohorts with adequate female representation.

EVAR has become the preferred treatment modality for iAAAs owing to its less invasive nature and favorable early outcomes [1,2,3,6,9,11,18,19,20,21,22,23]. However, concerns regarding long-term durability persist, as complications such as endoleaks and secondary interventions have been reported in up to 30% of patients within a decade [3]. In our previously published study on late EVAR conversion, patients requiring conversion to open repair demonstrated significantly increased perioperative morbidity and mortality, highlighting the substantial clinical burden associated with late EVAR failure [24]. These findings emphasize the importance of meticulous preoperative planning and detailed anatomical assessment. Although adherence to device-specific instructions for use (IFU) has been associated with improved outcomes in previous studies [25,26], our dataset did not include a formal analysis of IFU compliance; therefore, this aspect should be interpreted in the context of existing literature rather than as a direct finding of the present study. In our cohort, preoperative parameters such as aneurysm length, maximum diameter, total sac volume, and thrombus burden were significantly associated with the development of endoleaks and the need for secondary interventions. These results highlight the value of morphovolumetric assessment in predicting post-EVAR outcomes and support a more individualized risk stratification strategy rather than a uniform surveillance approach.

Volumetric sac analysis may provide a complementary assessment beyond conventional diameter-based measurements in detecting early aneurysm sac changes, particularly in patients with T2ELs, where subtle volumetric expansion may precede measurable diameter increase. Despite this advantage, maximum sac diameter remains the most widely used parameter in routine clinical practice due to its simplicity, rapid assessment, and reproducibility across centers. However, reliance solely on diameter measurements may underestimate clinically relevant sac progression, especially in cases with asymmetric or localized expansion. In contrast, volumetric analysis offers a more comprehensive evaluation of sac dynamics and may improve early risk stratification. With the development of automated and semi-automated imaging software, volumetric assessment is becoming increasingly feasible and time-efficient, suggesting that it may play a more prominent role in post-EVAR surveillance in the future, particularly for patients at higher risk of persistent endoleaks.

Endoleak subtype-specific analyses revealed distinct and clinically relevant risk profiles. Type Ia endoleak was significantly associated with advanced age, short proximal neck length, conical neck morphology, α-angle > 55°, presence of neck calcification, and neck thrombus (all *p* < 0.05). Also supported by previous studies that presence of more than one risk factor of those significantly effects development of Type Ia endoleak [27]. Type Ib endoleak was significantly correlated with peripheral arterial disease, aneurysm length > 133 mm, and the presence of iliac artery aneurysms (*p* < 0.05). Ongoing aneurysmal disease and the radial force of the endograft may also be factors influencing this type of endoleak. Type II endoleak demonstrated strong associations with dual antiplatelet therapy (*p* < 0.001), inferior mesenteric artery diameter > 3 mm (*p* < 0.05), the presence of ≥4 patent lumbar arteries (*p* < 0.05), increased thrombus-to-lumen ratio (TR/AO) (*p* < 0.05), and posterior thrombus localization (*p* < 0.05). Type III endoleak was significantly associated with larger aneurysm diameter, increased thrombus volume, iliac tortuosity, and iliac artery aneurysm presence (*p* < 0.05).

The observed association between dual antiplatelet therapy and endoleak development should be interpreted cautiously. In our practice, DAPT was preferentially prescribed in patients with external iliac artery extension, severe iliac tortuosity, or significant atherosclerotic disease. These anatomical and clinical features may independently reflect greater procedural complexity and higher endoleak risk. Therefore, residual confounding by indication is likely, and this study cannot establish a causal relationship between DAPT and endoleak development. Although impaired sac thrombosis remains a biologically plausible mechanism, the present finding should be regarded as hypothesis-generating.

The management of T2EL remains particularly challenging. Consistent with prior studies, our findings support a selective treatment approach based on aneurysm sac dynamics rather than endoleak presence alone. Persistent endoleaks associated with sac expansion, especially greater than 5 mm, should prompt intervention, whereas stable sacs may be safely observed. Endovascular treatment strategies for T2EL primarily include transarterial and translumbar embolization techniques, each with distinct advantages and limitations. Transarterial approaches are less invasive and suitable for anatomically favorable cases but may be limited by vessel tortuosity and incomplete nidus occlusion. In contrast, translumbar embolization allows direct access to the aneurysm sac and has demonstrated higher technical success and durability, particularly in cases with complex collateral anatomy or failed prior interventions. In our opinion, translumbar embolization should be considered the preferred approach in such complex cases, while transarterial embolization remains a valid first-line option in selected patients. In our cohort, no preemptive intervention was performed for T2ELs, and only two patients required post-EVAR intervention during follow-up. Despite advances in embolization techniques, recurrence remains a significant issue, often related to incomplete treatment of the endoleak nidus and collateral vessels. Therefore, effective management requires embolization of both inflow and outflow vessels in addition to the nidus itself, frequently utilizing combined embolic materials such as coils and liquid agents to improve long-term occlusion rates [26].

Technological advancements in endograft design have contributed to improved procedural outcomes. Devices incorporating active fixation mechanisms, such as EndoAnchors, have been shown to enhance proximal sealing and reduce the incidence of type I endoleaks, particularly in patients with hostile neck anatomy [2,7,28]. However, in our practice, EndoAnchor use has been limited, primarily due to additional procedural cost and resource considerations. Therefore, our approach continues to rely on careful preoperative planning and strict adherence to anatomical suitability criteria to achieve adequate proximal sealing. Nevertheless, type III endoleaks, often related to modular disconnection or graft material fatigue, remain a concern, particularly in large aneurysms and those with significant iliac tortuosity [11,20,21,22,29].

Type III endoleaks are closely related to device- and procedure-specific factors, including modular component overlap, component separation, graft fatigue, large aneurysm morphology, and iliac tortuosity. In large or elongated aneurysm sacs, persistent mechanical stress may increase the risk of modular instability over time [10]. Severe iliac tortuosity may further impair device alignment, reduce effective overlap, and increase conformational stress on modular junctions. These mechanisms may explain the observed association between larger aneurysm dimensions, iliac tortuosity, iliac aneurysm involvement, and Type III endoleak in the present cohort.

Imaging surveillance remains a cornerstone of post-EVAR management. Current guidelines from the Society for Vascular Surgery (SVS) and European Society for Vascular Surgery (ESVS) recommend structured follow-up protocols, typically incorporating computed tomography angiography (CTA) at 1, 6, and 12 months, followed by annual imaging [7,12]. Alternative modalities such as duplex ultrasound (CDUS), contrast-enhanced ultrasound (CEUS), and magnetic resonance angiography (MRA) may be utilized selectively, particularly in patients requiring reduced radiation or contrast exposure. It has been reported that approximately 0.5% of patients undergoing EVAR may develop radiation-induced malignancy over their lifetime, largely attributable to serial postoperative CT imaging [30]. This underscores the importance of tailoring follow-up protocols based on individual patient risk, aneurysm sac behavior and the presence or absence of endoleaks. In selected low-risk patients, alternative imaging modalities such as DUS or CEUS may reduce radiation exposure while maintaining adequate surveillance.

In our cohort, comparison of patients who underwent secondary intervention versus those who did not revealed a median survival of 86 months and 66 months, respectively. However, this difference was not statistically significant according to Kaplan–Meier analysis (log-rank *p* = 0.556). These findings suggest appropriate secondary interventions on time may mitigate the potential adverse impact of post-EVAR complications on long-term survival. The integration of volumetric analysis into follow-up protocols may further improve sensitivity for detecting clinically relevant changes.

This study has several limitations. First, its retrospective, single-center design introduces potential selection bias, residual confounding, and institutional variability in imaging and treatment strategies. In addition, because the retrospective database was constructed from patients who already fulfilled the final imaging and follow-up eligibility criteria, a formal screening flow diagram with precise exclusion counts for each criterion could not be generated; this represents an additional methodological limitation of the dataset. Second, although volumetric measurements were performed using standardized semi-automated software by experienced readers, formal interobserver and intraobserver reproducibility analyses were not performed. Therefore, the proposed volumetric thresholds, including the >164 cm^3^ cutoff, should be considered exploratory and require prospective external validation.

Third, the limited number of events in some endoleak subtypes, particularly Type Ia, Type III, and Type V, increases the risk of overfitting in subtype-specific analyses. Accordingly, these regression and survival findings should be interpreted as hypothesis-generating rather than definitive predictive conclusions. Fourth, the observed association between DAPT and endoleak may reflect confounding by indication, as DAPT was more frequently used in patients with complex iliac anatomy, external iliac artery extension, or advanced atherosclerotic disease; therefore, this association should not be interpreted as causal. In addition, the cohort was predominantly male, limiting evaluation of sex-specific outcomes, and overlapping endoleak subtypes could not be reconstructed consistently in all patients, which may affect subtype-specific interpretation. Finally, loss to follow-up in 10.4% of patients and COVID-19-related delays in surveillance imaging may have affected endoleak detection timing, time-to-reintervention estimates, and survival analyses. Larger multicenter studies with standardized imaging protocols and external validation are required before these thresholds can be used as clinical decision rules.

Advances in imaging technologies and computational modeling may further enhance individualized patient management. The endoleak management following EVAR requires a nuanced, patient-specific approach integrating anatomical characteristics, aneurysm sac dynamics, and procedural considerations. Rather than relying solely on endoleak classification, a comprehensive strategy incorporating morphovolumetric assessment and tailored intervention thresholds may improve long-term outcomes. Future multicenter prospective studies should validate morphovolumetric thresholds using standardized CTA protocols, reproducibility testing, and externally validated prediction models. Integration of automated segmentation tools, sex-specific anatomical assessment, and time-dependent sac remodeling analyses may further refine individualized post-EVAR surveillance strategies.

## 5. Conclusions

EVAR remains an effective treatment for infrarenal abdominal aortic aneurysms, although long-term outcomes are influenced by endoleaks, aneurysm sac behavior, and the need for secondary interventions. In this study, preoperative anatomical and morphovolumetric parameters were associated with post-EVAR endoleak development. Volumetric analysis may complement conventional diameter-based surveillance by identifying subtle sac remodeling, particularly in patients with type II endoleaks; however, the proposed thresholds remain exploratory and require external validation. Endoleak management should be guided by endoleak subtype, aneurysm sac behavior, and patient-specific anatomical risk factors. Type I and III endoleaks require prompt intervention, whereas type II endoleaks may be managed selectively according to persistence and sac enlargement.

## Figures and Tables

**Table 1:** Baseline and perioperative variables according to overall endoleak status.

Variable	No Endoleak (*n* = 283)	Endoleak (*n* = 100)	*p*-Value
Postoperative medication: ASA	195 (68.9%)	36 (36.0%)	<0.001
Postoperative medication: ASA + clopidogrel	55 (19.4%)	58 (58.0%)	<0.001
Postoperative medication: clopidogrel	18 (6.4%)	2 (2.0%)	<0.001
Postoperative medication: NOAC	15 (5.3%)	4 (4.0%)	<0.001
Preoperative aneurysm length, mm	125.2 +/− 17.9	135.7 +/− 19.8	<0.001
Preoperative D-dimer, ug/mL	2.6 +/− 3.0	3.7 +/− 3.5	0.006
Postoperative D-dimer, ug/mL	4.5 +/− 4.7	6.8 +/− 7.2	0.010
Preoperative aneurysm diameter, mm	62.4 +/− 10.4	66.6 +/− 12.4	0.003
Preoperative aneurysm volume, cm^3^	225.1 +/− 106.5	267.0 +/− 127.8	0.004
Preoperative thrombus volume, cm^3^	104.2 +/− 58.7	134.5 +/− 93.9	0.003

**Table 2:** Univariate logistic regression analyses with available OR and 95% CI.

Endpoint	Variable	Comparison/Scale	Univariate OR	95% CI	*p*-Value
Overall endoleak	ASA + clopidogrel	Yes vs. no	5.72	3.49–9.39	<0.001
Type Ia endoleak	Conical neck morphology/Delta A-B	>=3 mm vs. <3 mm	12.75	4.26–38.15	<0.001
Type Ia endoleak	Infrarenal neck–sac angulation	>60 deg vs. <=60 deg	12.11	4.22–34.70	<0.001
Type Ia endoleak	Neck thrombus	Present vs. absent	4.00	1.42–11.27	0.009
Type Ia endoleak	Neck calcification	Present vs. absent	23.57	6.51–85.34	<0.001
Type Ia endoleak	Neck length/C diameter	<=15 mm vs. >15 mm	3.48	1.21–9.96	0.020
Type II endoleak	ASA + clopidogrel	Yes vs. no	6.00	3.12–11.53	<0.001
Type II endoleak	IMA diameter	>3 mm vs. <=3 mm	18.37	8.97–37.62	<0.001
Type II endoleak	TR/AO ratio	Per 1% increase	0.97	0.94–0.99	0.005
Type II endoleak	Thrombus localization	Anterior vs. other	2.90	1.44–5.84	0.003
Type II endoleak	Patent lumbar arteries	>=4 vs. <4	6.48	1.97–21.36	0.002

**Table 3:** Multivariable logistic regression analyses for endoleak subtype-specific risk factors.

Endoleak Subtype	Variable	Adjusted OR	95% CI	*p*-Value
Type Ia	Delta A-B >= 3 mm	16.89	4.05–70.48	<0.001
Type Ia	Infrarenal neck–sac angulation >60 degrees	5.72	1.36–24.03	0.017
Type Ia	Neck thrombus present	4.22	1.03–17.22	0.045
Type Ia	Neck calcification present	21.28	4.45–101.70	<0.001
Type II	ASA + clopidogrel	9.23	3.93–21.70	<0.001
Type II	IMA diameter > 3 mm	17.54	7.34–41.91	<0.001
Type II	Preoperative TR/AO (%)	0.96	0.93–0.99	0.014
Type II	Anterior thrombus localization	2.98	1.16–7.66	0.023

## Data Availability

The data presented in this study are available on request from the corresponding author.

## Supplementary Materials

The following supporting information can be downloaded at: https://www.mdpi.com/article/10.3390/jcm15114300/s1.
