# Supplementary material for: Post-EVAR Endoleaks: A Morphovolumetric Approach to Prediction, Surveillance, and Management
Source: J Clin Med. 2026 Jun 2;15(11):4300. doi: 10.3390/jcm15114300 (PMC13257662; doi:10.3390/jcm15114300)
Supplement: Supplementary file 1 [file jcm-15-04300-s001.zip › jcm-4331996-supplementary.pdf]

Supplementary Table S1. ROC analysis of morphometric and morphovolumetric predictors

Outcome	Variable	Cutoff	AUC	95% CI	Sensitivity	Specificity	p-value
Overall endoleak	Preoperative aneurysm length	>133 mm	0.664	0.614-0.711	-	-	<0.001
Overall endoleak	Preoperative aneurysm diameter	>59 mm	0.616	0.565-0.665	-	-	<0.001
Overall endoleak	Preoperative aneurysm volume	>164 cm3	0.622	0.571-0.671	-	-	<0.001
Overall endoleak	Preoperative thrombus volume	>89 cm3	0.598	0.547-0.648	-	-	0.006
Secondary intervention	Preoperative aneurysm length	>132 mm	0.721	0.673-0.765	74.1%	62.9%	<0.001
Secondary intervention	Preoperative aneurysm diameter	>64 mm	0.622	0.572-0.671	55.6%	65.4%	0.004
Secondary intervention	Preoperative aneurysm volume	>189 cm3	0.641	0.591-0.689	40.7%	82.4%	0.001
Secondary intervention	Preoperative thrombus volume	>106 cm3	0.646	0.595-0.693	38.9%	88.2%	0.002
Secondary intervention	Preoperative TR/AO ratio	>51%	0.684	0.635-0.730	61.1%	75.7%	<0.001
